# MRI Findings of Endogenous Endophthalmitis as a Complication of Pneumococcal Meningitis

**DOI:** 10.5334/jbsr.4047

**Published:** 2025-09-23

**Authors:** Charlotte Steinier, Pierre-Antoine Poncelet

**Affiliations:** 1 Department of Intensive Care, Cliniques Universitaires UCL Mont-Godinne, Université catholique de Louvain, Belgium; 2 Department of Medical Imaging, Grand Hôpital de Charleroi (GHdC), Belgium

**Keywords:** endophtalmitis, meningitis, MRI, pneumococcal infection

## Abstract

*Teaching point:* Endophthalmitis is an uncommon but severe complication of meningitis that can be visualized on MRI.

## Case History

A 56‑year‑old female with a history of chronic alcoholism presented to the emergency department with fever and confusion. A diagnosis of Streptococcus pneumoniae meningitis originating from left sphenoidal sinusitis was confirmed by CT scan, laboratory tests, and lumbar puncture.

Within hours of admission, the patient developed pneumococcal sepsis, requiring intensive care unit admission. A few days later, left corneal edema and conjunctival hemorrhage appeared, raising the suspicion of endophthalmitis.

**Figure 1:**
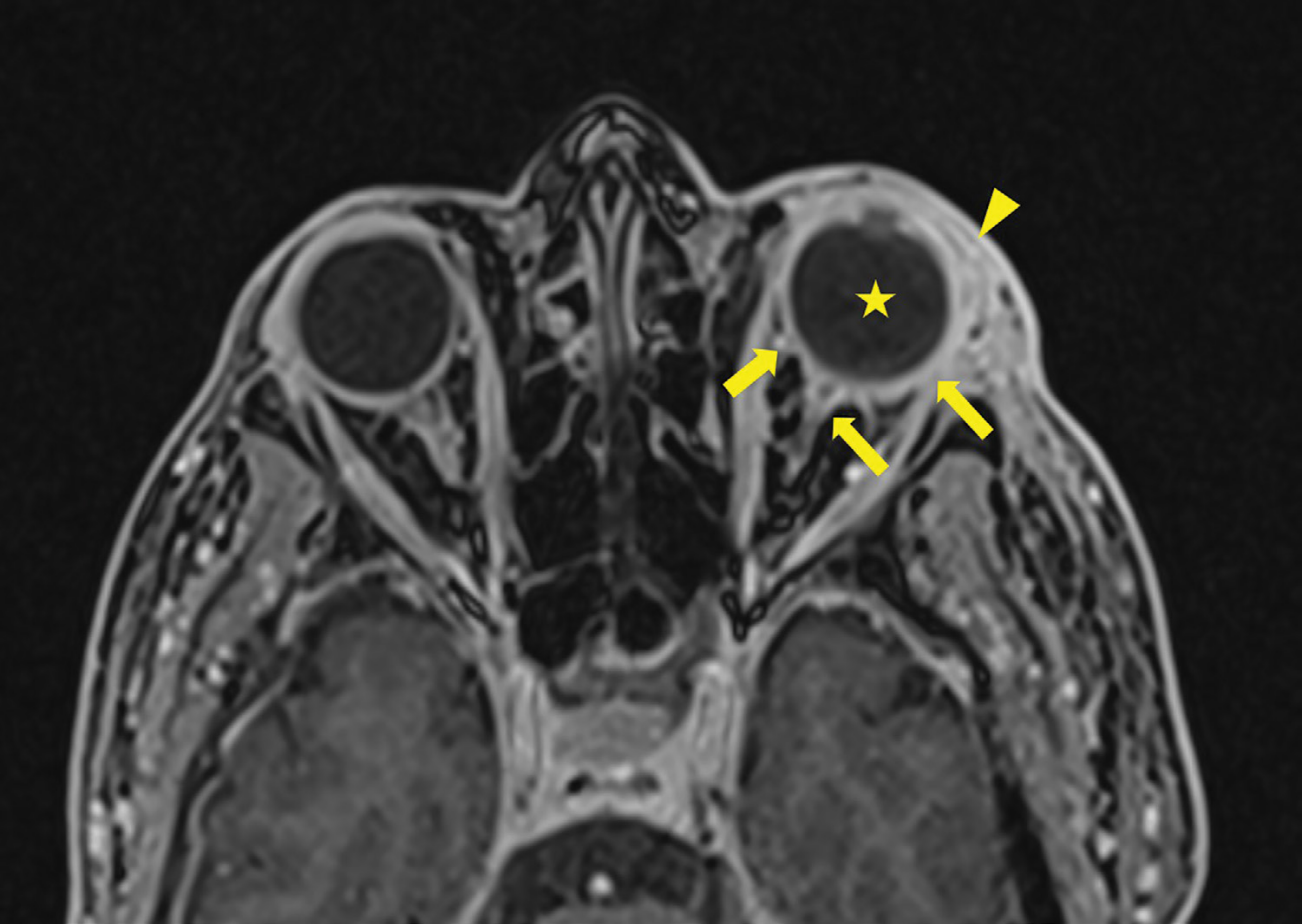
3D T1 Dixon Sequence showing pre‑septal enhancement (arrowhead) with an increase of the sclera enhancement as well as the junction between the left ocular globe and the optic nerve (arrows). Note the slight increase in T1 signal of the vitreous body (star).

**Figure 2:**
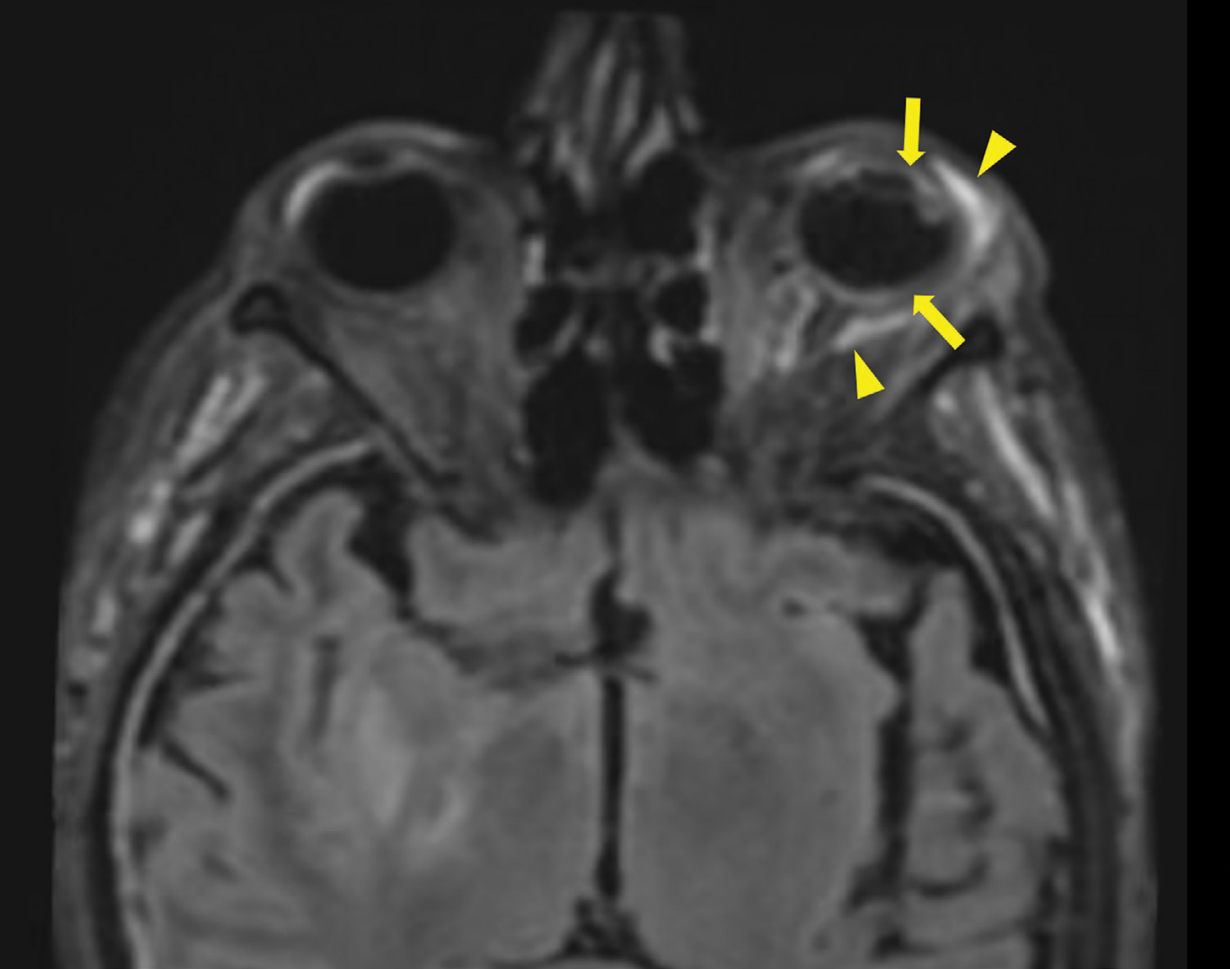
3D FLAIR Sequence with Fat saturation post contrast injection showing a pre‑septal and a perioptic hypersignal (arrowheads), a increase signal of the choroid and of the ciliary body. Also note the thickened dura mater in relation to the meningitis.

**Figure 3:**
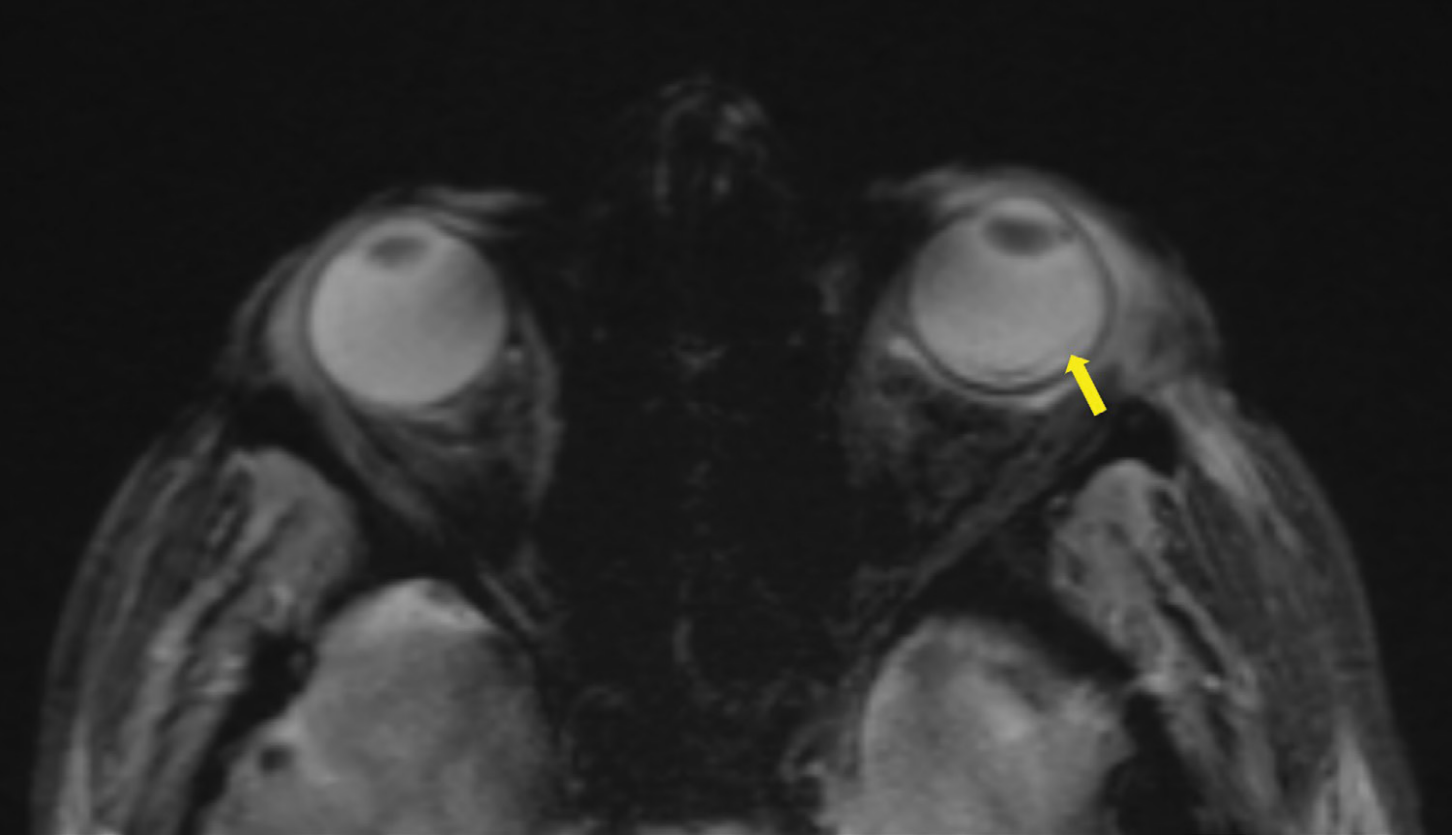
Axial T2 gradient echo sequence showing a retinal detachment with a slight linear hypoT2 signal (arrow).

After ruling out cavernous sinus thrombosis with a contrast‑enhanced CT scan, a brain MRI was performed, confirming the diagnosis of endophthalmitis. MRI showed pre‑septal orbital cellulitis, with pre‑septal enhancement seen on the 3D T1 Dixon sequence (Figure 1, arrowhead). This was associated with increased scleral enhancement and alterations at the junction between the left ocular globe and the optic nerve (Figure 1, arrow). Of note also the slight increase in T1 signal of the vitreous body (Figure 1, star). There was choroidal and peri‑septal inflammation with fat saturation after contrast injection, and in the 3D FLAIR sequence, showing a pre‑septal and peri‑optic hypersignal (Figure 2, arrowheads). Additionally, there was an increased signal in the choroid and ciliary body, as well as thickened dura mater, in relation to the meningitis (Figure 2). Axial T2 gradient echo sequences showed retinal detachment and inflammation around the distal portion of the optic nerve, with a slight linear hypoT2 signal (Figure 3, arrow).

Despite receiving intravenous and intraocular ceftriaxone therapy, the patient was completely blind in the left eye upon awakening. The meningitis was treated, and the patient underwent vitrectomy and ocular prosthesis placement.

## Discussion

Endophthalmitis is an infection of the eye’s internal structures [1]. It can be exogenous, due to microbial inoculation through an ocular breach, or endogenous, occurring in the context of septicemia when bacteria cross the blood‑ocular barrier from a distant infectious focus. Endogenous endophthalmitis is an uncommon infection that can, in rare cases, occur via neurogenic or meningeal spread along the optic nerve. It can occasionally arise as a complication of bacteremia.

This condition primarily affects immunocompromised or vulnerable individuals, although it may also occur in immunocompetent patients. While typically unilateral, bilateral involvement may occur. Clinical presentation usually includes a red, painful eye and sudden loss of visual acuity.

Although rare, this infection can lead to permanent blindness and requires prompt diagnosis and treatment.

## Conclusion

Endogenous endophthalmitis is a rare but potentially devastating condition. Rapid diagnosis and treatment are essential to maximize the preservation of visual function.

In intubated patients, regular ocular examination can be considered, particularly in the setting of prolonged bacteremia or a suspected deep‑seated infection. When performing cerebral MRI in the context of sepsis, radiologists and clinicians should pay particular attention to the orbital structures to detect possible signs of endophthalmitis and associated complications.
